# Dr. Myra Amanda Gillette

**Published:** 1913-05

**Authors:** 


Dr. Myra Amanda Gillette, Hahnemann of Chicago, 1886, died at her home in Medina, March 10, aged 59, of tuberculosis.

